# Medicines Optimisation Assessment Tool (MOAT): a prognostic model to target hospital pharmacists' input to improve patient outcomes. Protocol for an observational study

**DOI:** 10.1136/bmjopen-2017-017509

**Published:** 2017-06-14

**Authors:** Cathy Geeson, Li Wei, Bryony Dean Franklin

**Affiliations:** 1 Pharmacy, Luton and Dunstable University Hospital, Luton, Bedfordshire, UK; 2 UCL School of Pharmacy, London, UK; 3 Centre for Medication Safety and Service Quality, Imperial College Healthcare NHS Trust, London, UK

**Keywords:** Epidemiology, Risk management, Adverse events

## Abstract

**Introduction:**

Medicines optimisation is a key role for hospital pharmacists, but with ever-increasing demands on services there is a need to increase efficiency while maintaining patient safety. The aim of this study is to develop a prognostic model, the Medicines Optimisation Assessment Tool (MOAT), which can be used to target patients most in need of pharmacists' input while in hospital.

**Methods and analysis:**

The MOAT will be developed following recommendations of the Prognosis Research Strategy partnership. Using a cohort study we will prospectively include 1500 adult patients from the medical wards of two UK hospitals. Data on medication-related problems (MRPs) experienced by study patients will be collected by pharmacists at the study sites as part of their routine daily clinical assessment of patients. Data on potential risk factors such as polypharmacy, renal impairment and the use of 'high risk' medicines will be collected retrospectively from the information departments at the study sites, laboratory reporting systems and patient medical records. Multivariable logistic regression models will then be used to determine the relationship between potential risk factors and the study outcome of preventable MRPs that are at least moderate in severity. Bootstrapping will be used to adjust the MOAT for optimism, and predictive performance will be assessed using calibration and discrimination. A simplified scoring system will also be developed, which will be assessed for sensitivity and specificity.

**Ethics and dissemination:**

This study has been approved by the Proportionate Review Service Sub-Committee of the National Health Service Research Ethics Committee Wales REC 7 (16/WA/0016) and the Health Research Authority (project ID 197298). We plan to disseminate the results via presentations at relevant patient/public, professional, academic and scientific meetings and conferences, and will submit findings for publication in peer-reviewed journals.

**Trial registration number:**

NCT02582463.

**Strengths and limitations of this study**

- The Medicines Optimisation Assessment Tool (MOAT) will be the first evidence-based prognostic model to identify hospitalised patients at risk of moderate or severe medication-related problems in order to permit targeting by pharmacists.
- The study will include adult patients of all ages admitted to all types of medical wards (general, emergency and elderly medicine), so will be representative of patients routinely admitted to hospital medical wards.
- The method and analysis plan are based on the Prognosis Research Strategy, Transparent Reporting of a multivariable prediction model for Individual Prognosis Or Diagnosis (TRIPOD) and CHecklist for critical Appraisal and data extraction for systematic Reviews of prediction Modelling Studies (CHARMS) recommendations for prognostic research.
- The study is observational, which may be subject to reporting bias and missing data.
- Only two hospitals will be included in the study; further validation, impact and implementation studies will be needed to determine whether the MOAT could be successfully employed in new settings.

## Introduction

Medicines play a crucial role in maintaining health and are the most common intervention in healthcare. However, in the UK, as elsewhere, there is a growing body of evidence that there is a need to improve medicines use.^1–6^ This includes the Francis and Berwick reports,^1 2^ which call for a number of actions to improve patient safety and reduce avoidable harm.

Historically, adverse drug events have been the focus of studies of medication-related harm,^7^ but problems can also result from suboptimal medicines use, such as ineffective treatments or subtherapeutic doses. It is estimated that only 4%–21% of patients in primary care receive optimum benefit from their medicines,^8^ and it has been suggested that research efforts should also identify patients with unrealised benefits.^9^ A term that encompasses both aspects is medication-related problems (MRPs), defined as all circumstances involving a patient’s drug treatment that actually, or potentially, interfere with the achievement of an optimal outcome.^7 10–12^ This also shifts the focus from ‘medication-related harm’ to ‘medicines optimisation’, which can be described as the safe and effective use of medicines to enable the best possible outcomes.^5^ Medicines optimisation is high on the English national agenda, with guidance issued by the Royal Pharmaceutical Society and the National Institute for Health and Care Excellence.^4 5^

Medicines optimisation is a key role for pharmacists,^13–16^ and a number of systematic reviews conclude that addition of clinical pharmacy services to the care of hospital inpatients improves quality, safety and efficiency of patient care.^10 17 18^ Ideally, pharmacists would see every patient daily, but medicines optimisation is not the only goal for hospital pharmacy services in England.^13 19^ Other service developments are required, such as delivery of 7-day services^20^ and the Hospital Pharmacy Transformation Programme, as set out in the recent review by Lord Carter on improving productivity and performance in English National Health Service (NHS) acute hospitals.^21^ Owing to financial challenges that face the NHS, these developments often have to be achieved within existing funding through increased efficiency and innovation.^5 22 23^ There have therefore been calls from international government organisations and professional bodies for effective ways for pharmacy services to target patients most in need.^15 24–28^

Clinical prioritisation has been proposed as a way to permit pharmacy services to focus on the greatest need and where clinical pharmacy input is likely to have greatest impact. This requires a method to triage patients to assign ‘pharmaceutical acuity’.^28 29^ There are recognised risk factors for MRPs, for example polypharmacy, renal impairment and the use of ‘high risk’ medicines,^30^ but to target patients appropriately pharmacists need to be able to apply this knowledge effectively and consistently within their routine clinical practice.

Predicting clinical risk is well established in medicine. Tools such as cardiac-risk calculators and the Waterlow score (to assess the risk of pressure ulcers) are both used daily across the NHS.^31 32^ Prediction tools to identify hospitalised patients at risk of adverse medication-related outcomes have been developed,^33–41^ but the majority identify patients at risk of adverse drug reactions,^34 35^ adverse drug events^36^ or medication errors,^37^ rather than MRPs, or are based on ‘expert opinion’ rather than statistical determination.^38–41^

Interest in prediction research (also known as prognosis research) has developed rapidly in recent years. It involves use of statistical methods to predict future health outcomes among people with a given baseline health status, and therefore has potential to inform clinical decision making, improve patient care and make healthcare more efficient.^42 43^ Prognostic modelling is one component of prognosis research, in which multiple risk (prognostic) factors are statistically combined to predict future clinical risk for an individual patient.^44^ However, many published prognostic model studies have been criticised in terms of methodological shortcomings, limiting their reliability and applicability,^44 45^ as well as poor reporting, which limits the ability to effectively assess the risk of bias.^43 46^ Both problems ultimately limit the usefulness of the prognostic models. The perceived inadequacies in prognostic model research prompted the recent publication of recommendations for prognosis research by the Prognosis Research Strategy (PROGRESS) partnership,^42 44 47 48^ together with specific guidelines for reporting^43 46^ and critically appraising^49^ prognostic model research.

This study aims to address a current gap in the evidence base: the development of a methodologically sound prognostic model to target hospital patients most in need of pharmacists’ input based on their risk of MRPs. The purpose of publishing this study protocol is to expand on the details already publicly available^50^ to clearly specify a priori the outcome measure, prognostic factors and analysis plan. This is intended to protect against both data-driven model development (associated with overoptimistic model performance) and selective reporting. The study method is informed by the PROGRESS guidelines, and the protocol includes the key elements proposed by Peat *et al* 43 for inclusion in prognostic model protocols, and follows the Transparent Reporting of a multivariable prediction model for Individual Prognosis Or Diagnosis (TRIPOD) Statement guidance^46^ and the CHecklist for critical Appraisal and data extraction for systematic Reviews of prediction Modelling Studies (CHARMS) checklist^49^ in terms of the level of detail provided. This is to ensure provision of sufficient information to permit full assessment of risk of bias and applicability.

The aim of this study is to develop a prognostic model, the Medicines Optimisation Assessment Tool (MOAT), to identify adult patients at highest risk of preventable, moderate or severe MRPs during admission to a UK medical ward. Additional objectives are to assess the MOAT’s content validity, feasibility of use, potential efficiency savings and the potential clinical risk associated with false-negative predictions. The proposed purpose of the MOAT is to permit appropriate targeting of patients by pharmacy staff in order to reduce risks, improve patient outcomes and increase efficiency of hospital clinical pharmacy services, thereby supporting delivery of national targets related to patient safety, medicines optimisation and service provision.

## Methods and analysis

### Design

This is a prognostic model development study that aims to select candidate predictors (the potential prognostic factors) and combine them into a multivariable model using logistic regression. Internal validation (bootstrapping) will be used to evaluate the performance of the model and permit adjustment for optimism. The MOAT will be developed using a prospective cohort study involving adults admitted to the medical wards at two UK hospitals in South East England. Two study sites, Hospitals A and B, were chosen to increase generalisability of the MOAT as they have markedly different patient demographics. It is anticipated that the study will be completed by April 2018.

### Eligibility criteria

Patients aged 18 years old or over will be selected by means of being consecutive admissions to the medical wards (general, emergency and elderly medicine) at the two study sites during the study period. Patients admitted to other specialties such as surgery, maternity and paediatrics will be excluded due to potential differences in the prevalence/type of MRPs in these patient groups.

Patients will be excluded if:

- their admission is for investigation only (as changes to medication will be minimal)
- they are not prescribed any medication during the admission
- their entire admission is outside of core pharmacy working hours (ie, 09:00–17:00 Monday–Friday) as these patients are unlikely to receive review by a clinical pharmacist
- their prescription is not reviewed by a clinical pharmacist during the admission (eg, a patient who is present on a study ward during core pharmacy working hours but discharged before a clinical pharmacist is able to review his/her medication).

### Outcome

The outcome of interest for the prognostic modelling will be MRPs that are at least moderate in severity and preventable. Research has shown that a significant proportion of hospitalised patients will experience MRPs (eg, Blix *et al* 51 reported a rate of 81%), many of which are of limited clinical significance. We will therefore use moderate or severe MRPs as this will enable the MOAT to target patients most in need of pharmacist input in terms of risk of medication-related harm or suboptimal use. It will also ensure that the MOAT is clinically relevant and feasible to implement in terms of pharmacists’ workload. Similarly, only MRPs considered as ‘preventable’ will be considered to ensure that the MOAT identifies patients with MRPs that are amenable to pharmacist intervention either directly or through discussion with prescribers.

The definition for MRPs that will be used is ‘all circumstances involving a patient’s drug treatment that actually, or potentially, interfere with the achievement of an optimal outcome’.^7 10–12^

All MRPs will be classified for descriptive reporting purposes. As there is no universally accepted classification system and perceived deficiencies with some systems, we will use the aggregated classification system recently developed by Basger *et al*.^52^ This provides a comprehensive classification system based on the causes of MRPs, thereby preventing any potential confusion between MRP ‘causes’ and ‘outcomes’.

MRP data will be identified and recorded by pharmacists at the study sites as part of their routine daily clinical assessment of patients. They will record data on all MRPs identified personally or through discussion with other healthcare professionals. The hospital incident reporting systems will also be reviewed to check for any additional significant MRPs that are not identified by pharmacy staff. Following training on the use of Basger’s aggregated classification system, pharmacists will be asked to classify each MRP at the point of identification.

MRP data will be collected for all study patients from admission to discharge from hospital, or the date the study closes (2 weeks after inclusion of the final study patient), whichever occurs sooner. A study close date will be used to facilitate practicality in terms of data collection, while permitting data to be collected from admission to discharge for the majority of study patients (as the mean length of stay at the study sites is approximately 6 days).

Previous research into the detection of prescribing errors, a subset of MRPs, has shown that the observed incidence is extremely dependent on the method of detection.^53^ We have chosen to use prospective identification by pharmacists for this study because (1) the purpose of the study is to develop a prognostic model for MRPs that can be identified during routine clinical practice by pharmacists; (2) it will permit the identification/inclusion of MRPs that are not routinely recorded in medical notes, such as potential prescribing or administration errors that are intercepted; and (3) it will permit the MRPs to be identified by staff personally involved in the care of the study patients, increasing clinical and practical relevance.

It is acknowledged that a limitation will be the possibility of incomplete data due to pharmacy staff being required to complete this work in addition to other routine duties. To minimise this, the principal investigator will work closely with the study sites to ensure that data collection occurs at an optimal time in terms of staffing levels and workload. Staff involved in MRP data collection will also be provided with initial training to improve the consistency and reliability of data collection. The principal investigator will review all data collection forms daily and seek clarification where needed, and provide the pharmacists with ongoing fidelity training.

We also recognise that identification of MRPs may vary depending on the knowledge, experience and skills of the pharmacists collecting data. To quantify this potential variability, a simulated ‘MRP identification assessment exercise’ will be developed and used in a training scenario. Each simulated MRP will be treated as having a binary outcome in terms of whether or not it is identified by each pharmacist. Fleiss’ kappa will be used to calculate the level of agreement between pharmacists.

In prognostic research it is recommended that the outcome is assessed while blinded to the candidate predictors (potential prognostic factors) to prevent bias.^49 54 55^ In this study it will not be possible to blind the pharmacists collecting the outcome data to the patient’s clinical information (such as age, diagnosis, laboratory results and so on) as this information will form part of their clinical assessment of the patient. Despite this, the pharmacists collecting the outcome data will not know which factors will be used as candidate predictors in order to minimise the potential for this information to influence their outcome assessment.

Following anonymisation to maintain patient confidentiality and blinding, each potential MRP will be assessed by an expert panel. Agreement will be reached by consensus on whether it is a true MRP, and then confirmed MRPs will be assessed for severity. The expert panel will comprise the principal investigator, a hospital pharmacist, a senior nurse and a consultant physician. Once MRPs have been confirmed, the panel will assess each MRP for severity. As no established grading for MRPs is available, severity will be classified using a validated visual analogue scale for medication errors,^56^ used previously for this purpose by Rashed *et al.* 57 MRPs will be scored independently in terms of potential patient outcomes on a scale of 0–10, where 0 represents a case with no potential adverse effect on the patient and 10 a case that would result in death. The mean score for each MRP from the panel members will be used as an index of severity, with a score of less than 3 being considered as a minor outcome (very unlikely to have an adverse effect), a score of 3–7 will be considered as moderate (likely to cause some adverse effects or interfere with therapeutic goals, but very unlikely to result in death or lasting impairment), and a score of greater than 7 will be considered to be a severe outcome (likely to cause death or lasting impairment).

No established grading system for MRP preventability is available; therefore, we considered two possible methods: the criteria provided by Schumock and Thornton,^58^ and the ‘P Method’.^59^ We concluded that both methods were developed for adverse drug reactions, most of which are unpreventable, whereas the majority of MRPs are inherently preventable. Neither method was therefore appropriate for the present study. Pharmacists will therefore be asked to review each MRP at the point of identification to assess whether it was preventable, expressed as a dichotomous variable of yes or no. The principal investigator will then perform a second check of all MRPs to ensure consistency. To prevent ‘judgement drift’ a ‘case law document’ will be used.^60^

### Candidate predictors

Candidate predictors are the variables that predict the prognostic outcome. These can include patient demographics, clinical history, physical examination, disease characteristics, test results and treatments used.

When choosing the potential candidate predictors, various recommendations have been made:

- Predictors already reported as prognostic should be included.^61 62^
- The selection should be informed by clinical understanding (ie, expert opinion) to ensure the list is comprehensive and clinically relevant.^45 62^
- Where predictors are highly correlated (eg, weight and body mass index), only one should be selected.^62^
- Potential confounders (ie, a variable that may be associated with another predictor and the outcome) should be included to permit these to be accounted for during analysis.^63^
- Use of predictors that occur infrequently can lead to inaccurate results.^62 64^
- Candidate predictors should be:
  - available at the time when the model is intended to be used.^54^
  - clearly defined, standardised and reproducible (to enhance generalisability and applicability of study results to practice).^54^
  - have minimal measurement error (as this may dilute their prognostic value).^49^

A review of the published literature identified 59 possible predictors, but substantial variations were found between studies in terms of the strength of evidence for each predictor. This is potentially due to significant differences in study design, and the outcome measure used (namely adverse drug events, adverse drug reactions, prescribing errors and MRPs). Twenty-seven of the potential predictors were selected, based on the strength of published evidence in addition to the criteria stated above, for inclusion in a survey to obtain expert opinion from healthcare professionals and patient/public representatives. The survey was administered during April–June 2016, and a total of 247 responses were received. The results showed that the majority of the potential predictors (23 of 27) were considered ‘important’ or ‘very important’. In addition, a significant number of additional predictors (59) were suggested.^65^

When developing a prognostic model, it is necessary to limit the number of candidate predictors used to prevent ‘overfitting’ or ‘underfitting’. Both can lead to poor performance when the model is used in an independent data set.^55^ One method to reduce the number of candidate predictors is to base the selection on the univariable association between each predictor and the outcome. This is not recommended as it results in overfitting due to selection bias,^61^ and can lead to predictors being wrongly excluded from the model due to the fact that the association may only become significant after adjustment for the other predictors. It is recommended that the candidate predictors are selected a priori.^47 64^ We have therefore chosen to preselect the candidate predictors for development of the MOAT (table 1) using the recommendations above. An additional consideration was the selection of predictors that form part of standard clinical data sets. This was to increase the reliability of the data and minimise the potential for missing data, and to enable the MOAT to be readily incorporated into clinical practice without the need for additional tests/measurements.

**Table 1:** Preselected candidate predictors for the Medicines Optimisation Assessment Tool

Variable	Details/categories	Type of measurement	Number of variables*
**Demographic**			
Age	Age at admission to hospital (in years)	Continuous numeric	1
Socioeconomic status	Based on the English indices of deprivation 2015 (Index of Multiple Deprivation Rank)	Continuous numeric	1
**Patient-related**			
Previous allergy/adverse drug reaction	Yes/No	Binary	1
Body mass index	First documented result following admission	Continuous numeric	1
Number of hospital admissions	Number of admissions to the study hospital in the previous 6 months	Continuous numeric	1
Primary diagnosis	Categorised by ICD-10 coding: Endocrine Nutritional and metabolic diseases Diseases of the circulatory system Diseases of the respiratory system Diseases of the digestive system Diseases of the genitourinary system Other (all other diagnoses combined)	Nominal categorical	6
Number of comorbidities	From hospital clinical coding data (ICD-10 codes)	Continuous numeric	1
History of dementia	From hospital clinical coding data (ICD-10 codes)	Binary	1
**Medicines-related**			
Number of medicines prescribed	Number of ‘regular’ medicines prescribed on the first full day of admission to hospital (ie, excluding ‘when required’ and ‘once only’ medicines, dietary products, non-medicated topical products (eg, emollients), wound dressings)	Continuous numeric	1
Use of ‘high risk medicines’	Prescribed as a ‘regular’ medicine during the hospital admission: Anticoagulants/direct oral anticoagulants Therapeutic heparin Antidiabetic medication Opiates (excluding codeine, tramadol and dihydrocodeine) Aminoglycosides and glycopeptides Antibiotics (excluding aminoglycosides and glycopeptides) Theophylline and aminophylline Epilepsy medicines Antipsychotics Immunosuppressants (excluding corticosteroids) Cytotoxics Lithium Antiarrhythmics Antidepressants Other (clozapine, antiretrovirals, medicines for Parkinson’s disease)	Binary (for each group)	15
Parenteral administration route	Administration of one or more regular medicines via the parenteral route (intravenous, intramuscular, subcutaneous) during the hospital admission (excluding prophylactic low molecular weight heparins, fluid replacement therapy)	Binary	1
**Laboratory results**			
Renal function	Creatinine clearance calculated using the Cockcroft-Gault equation (using first documented results following admission)	Continuous numeric	1
Liver disease	Liver disease defined as ALT/ALP and/or bilirubin ≥3 times normal range and/or documented liver disease Laboratory results will be the first documented results following admission Documented liver disease will be established from hospital clinical coding data (ICD-10 codes)	Binary	1
Serum albumin	First documented result following admission	Continuous numeric	1
Serum potassium	First documented result following admission	Continuous numeric	1
Serum sodium	First documented result following admission	Continuous numeric	1
White cell count	First documented result following admission	Continuous numeric	1
Platelet count	First documented result following admission	Continuous numeric	1
Total number of variables*			37

*Number of variables in relation to calculating the ‘events per variable’.

ALP, alkaline phosphatase; ALT, alanine aminotransferase; ICD, International Statistical Classification of Disease and Related Problems.

All data on the candidate predictors will be collected retrospectively. Data will be obtained from the information department at the study hospitals where possible, including demographic, diagnostic and comorbidity data. Laboratory data will be extracted manually from the electronic reporting system used at both hospitals. The remaining data will be extracted manually from patient medical records. Hospital A has electronic medical and prescribing records; Hospital B has paper-based systems. Manual data extraction of laboratory data will be performed by a single data analyst at each study site, independently of the research team. Data from the patient medical records will be collected by the independent data analyst at Hospital A, but due to the use of paper-based systems at Hospital B and the need to read handwritten prescriptions, these data will be extracted by the principal investigator at Hospital B. All manually extracted data will be entered directly into an electronic database. All data will be recorded as reported, with no categorisation of continuous data.

In prognostic research it is recommended that data on candidate predictors is collected blind, in terms of knowledge of the outcome and other predictors.^49 55^ This is particularly important when subjective judgement is required as it prevents the assessment being influenced, which could artificially increase the associations between the predictors and outcomes. Full blinding will not be possible for this study as the independent data analysts and the principal investigator will not be blinded to all other predictor data, and the principal investigator will not be blinded to the MRP status. It is anticipated that this will have minimal impact on the accuracy of data collection as all candidate predictors selected for this study are objective measurements that are independent of observer interpretation; therefore, subjective judgement is not required. In addition all candidate predictors will be recorded contemporaneously during the admission as part of routine care/documentation, therefore without knowledge of the MRP status. To identify any possible bias, the principal investigator will perform a double check on data entry. This will involve a double check on a randomly selected 10% sample of the 1500 study patients. Sixteen data items will be checked for each of these 150 patients, giving a total of 2400 data items. The accuracy will be calculated as the percentage of data items recorded correctly. Data entry will be refined if necessary.

#### Sample size calculation

Sample size is often calculated based on significance testing (power calculations), but this is not straightforward for prognostic modelling studies as there is often not a clear ‘measure of effect’ to power the research. An alternative method is to calculate the sample size based on the desired precision of a sample estimate.^55^ An alternative approach that is commonly used is the ‘rule of thumb’ of '10 events per variable’ (EPV).^66^ This method requires the sample size to be based on the prevalence of the outcome measure and the number of candidate predictors that will be used in model development.^49 54 55 66^ Although there is debate over the optimal number of EPV, with recognition that ‘the rule of 10 or more EPV is not a well-defined bright line’,^64^ there is agreement that models developed with less than 10 EPV need to be interpreted with caution.^55 64^ The reason for the potential problem with using less than 10 EPV relates to the reliability of the model when used in a new group of patients. If a model is too closely adapted to the developmental data, it can reflect associations between the candidate predictors and outcome which are due to chance rather than true associations, known as ‘overfitting’ or ‘optimism’.^49 55 61^

For this study the sample size has been dictated by practical considerations (funding, time available and accessibility of data at the study sites), resulting in the capacity to include 1500 (1000 from Hospital A and 500 from Hospital B), plus an additional 10% per site to allow for exclusion of patients who do not meet the eligibility criteria. We have therefore used both the precision and EPV methods to consider the adequacy of this sample size, based on an estimation of the outcome prevalence in the study population.

The outcome of interest for this study is moderate or severe, preventable MRPs in hospitalised UK patients. No estimate for the prevalence of this outcome currently exists, but Blix *et al* 51 (Norway 2004) reported that 81% of 827 hospitalised patients experienced an MRP, with approximately half of all MRPs classified as ‘extremely important’ or ‘major’ in terms of clinical significance (preventability not reported). To establish the prevalence of the outcome in the study population, we carried out pilot work involving 200 patients, and found that 39% (95% CI 32% to 45%) experienced at least one moderate or severe, preventable MRP (using the proposed visual analogue scale for medication errors and a severity score of 3 or more). Although this is consistent with Blix’s work, we recognise that our estimate is based on a small sample of patients (200). In addition the MRPs were severity rated by three members of the expert panel rather than the four proposed for the main study. We have therefore chosen to use the lower CI limit as an estimate of the outcome prevalence, that is, 32%. Given an anticipated outcome prevalence of 32% and a sample of 1500 patients, we anticipate identifying 480 patients with at least one moderate or severe preventable MRP.

To consider the adequacy of the sample size using the precision method, we first established acceptable target sensitivity for the MOAT by including a question in our survey of healthcare professionals and patient/public representatives, detailed above. We proposed a target sensitivity of 90% and asked survey respondents if this was acceptable. This sensitivity was selected based on previous research to develop a ‘clinical decision rule’ to identify emergency department patients at risk of adverse drug events.^67^ Hohl *et al* 67 used a target sensitivity of 90% as this was deemed acceptable by emergency physicians and considered feasible for implementation in terms of workload for pharmacists. A total of 237 responses were received for this question: 189 (80%) answered that 90% was an acceptable target, 21 (9%) answered no and 27 (11%) were ‘unsure’. As a result we concluded that 90% is an acceptable target for sensitivity. Given the anticipated number of study outcomes and a target sensitivity of 90%, this will permit the precision of the sensitivity to be estimated with 95% CIs of ±3%, which we consider to be an acceptable level of precision in terms of clinical usefulness of the MOAT.

For the EPV method our aim would be to have at least 10 events for every variable used in model development. Given the estimate of 480 outcome events, that is to say patients with at least one moderate or severe preventable MRP, this would permit the inclusion of 48 ‘variables’ in model development. The number of variables includes all proposed candidate predictors, interactions examined (ie, where a candidate predictor has a different association with the response depending on the value of a third variable), transformations for continuous predictors (which permits modelling of non-linear predictors) and indicator variables for categorical predictors. We do not hypothesise any interactions a priori. We will explore any potential interactions during the analytical stage to establish whether there are associations that may lead to a better understanding of the final model, but recognise the risk of overfitting caused when numerous interactions are examined, with only the strongest included in the model.^55^ Similarly, we will not know whether transformations are required until we examine the linearity of the continuous predictors.

Table 1 shows the total number of variables that will be used for each candidate predictor (including the indicator variables, which are the artificial variables used to represent distinct groups within a categorical variable). We propose using 37 variables to develop the MOAT, resulting in 13 EPV, given that no interactions or transformations are required.

### Data analysis plan

#### Descriptive analysis

Descriptive information about the sample population will be provided. This will include the distribution of the relevant characteristics of study patients, including demographic data, the distribution of candidate predictors, the ranges of continuous predictors and the amount of missing data (with possible reasons for the missingness). This is to permit an assessment of the context, case mix and setting of the study; the ranges of continuous predictors that are compatible with the MOAT; and the potential impact of any missing data. Descriptive information will also be provided on the proportion of patients with MRPs, including the severity and preventability. We will also report the outcome frequency (namely patients with at least one MRP that is moderate or severe and preventable) across the predictor categories.

#### Statistical analysis

The data analysis plan has been developed prior to any analysis of the study data to prevent the potential for data-driven model development and type 1 errors.^47^ The study method is informed by the recommendations of the PROGRESS partnership^42 44 47 48^; however, it is recognised that a prognostic research protocol cannot be a ‘rigid blueprint’. Peat *et al* 43 state that it is neither possible nor desirable to prespecify all analysis. We therefore acknowledge that it may be necessary to modify the analysis plan or carry out additional analyses in light of new findings. Analysis will be conducted using SPSS V.24.

#### Missing data

Complete-case analysis can lead to selection bias^55 68^ and loss of statistical power/precision.^61^ We will therefore examine missing data to establish the missingness mechanism, and if we can establish that the data are ‘missing at random’ we will use multiple imputation (using ≥20 imputed data sets) to impute the missing values. We will then carry out complete-case analysis and compare the results with those from multiple imputation to assess for potential bias due to data that are ‘missing not at random’.

#### Candidate predictor handling

We will first review the measurement reliability of each candidate predictor and consider excluding any that are found to be unreliable.^55^ We will also consider if there are any closely related predictors that may need to be combined, or one excluded from the analysis.^55^ All candidate predictors that are measured as continuous variables will be analysed on their continuous scale, that is we will not dichotomise or use categorisation as this can lead to optimistic model performance.^47 49 55 61^ We have chosen to treat liver disease as a binary variable (see table 1), but this is because of the variation in liver function tests dependent on the type and stage of disease, and to be consistent with pharmacy prioritisation tools currently in use in the UK.^28^ Individual continuous predictors will be examined to identify/investigate unexpected values in order to establish if they are recording errors or true outliers. We will also check for linearity of continuous predictors and use appropriate data transformation if required.^69^ For categorical variables we will review the number of patients within each group and consider the need to collapse groups if there are insufficient patients to permit robust modelling.^61^

#### Model building

We will use multivariable logistic regression modelling to develop the MOAT. This has been chosen as the outcome is binary, and all participants will be followed up to the end of the study period. The aim is to produce a parsimonious model to increase clinical applicability while retaining reasonable predictive performance.

Backwards elimination will be used to reduce the set of candidate predictors. The Akaike Information Criterion (AIC) will be used to exclude predictors. We chose to use the AIC due to the relatively small data set (hence relatively larger p values for the predictors), as it is less likely to result in underfitting than alternative methods.^69^

#### Adjusting for optimism

The predictive performance of prognostic models is overestimated when assessed using the same sample data used in development (known as the apparent performance), simply because the model has been optimised for that data. To account for this ‘optimism’, we will assess the predictive performance of our original model using bootstrap validation. Steyerberg^69^ advises that 100–200 bootstraps may be sufficient; therefore, we plan to use 200, but will increase this if it is needed to achieve a stable estimate. We can then use this to calculate a ‘shrinkage factor’, which will be used to adjust our original model to produce the final model/MOAT.

#### Creating a simplified scoring system

The final prognostic model will be used to develop a simplified scoring system or ‘clinical decision rule’. These differ from prognostic models in that they indicate a specific course of action, rather than simply providing an estimate of risk, and have the advantage that they are simpler to use in clinical practice as they do not require complex calculations.

We will use the method developed by Sullivan *et al* 70 to convert the regression coefficients from the final prognostic model into a score. We will then create ‘risk groups’ (high, medium and low) based on the scoring system.

The development of the scoring system for the MOAT will require categorisation of the continuous predictors and the selection of appropriate cut-off points for the risk categories; we will therefore seek input from clinical experts to ensure that the grouping is clinically practical and appropriate.

#### Assessing model performance

We will assess the predictive performance of the final prognostic model using calibration (agreement between observed and expected predictions) and discrimination (the ability to differentiate between those who do or do not experience the outcome event). The calibration will be presented as a graph of the predicted risk of experiencing a preventable, moderate or severe MRP versus the observed risk in the study sample. Discrimination will be reported as the area under the receiver operating characteristic curve.

The predictive performance of the simplified MOAT scoring system will be reported using the classification measures: sensitivity and specificity.

### Future plans

The intention of this research is to develop a prognostic model with potential to be adopted widely into clinical practice. This requires the MOAT to be clinically credible, accurate, generalisable and clinically effective in improving decision making and patient outcomes.^71^

If the initial research is successful in producing a model with good predictive performance, we plan to conduct further research to assess content validity, feasibility of use, potential efficiency savings and any potential clinical risk to patients through use of the MOAT due to false-negative predictions.

Extensive external validation, involving prospective validation in a new cohort, will also be required to further assess accuracy and generalisability before routine use of the MOAT could be recommended.^44^ External validation will also provide opportunity to refine the MOAT in terms of improving the accuracy such as by updating the model^44 72^ and/or simplifying the scoring system.

Following external validation we also plan to carry out implementation and impact studies to establish whether the MOAT has advantages over current practice, is compatible with (and can easily be incorporated into) practice, has the potential to change pharmacists’ behaviour, has a positive impact on patient outcomes and is cost-effective. We would also investigate the possibility of incorporating the MOAT into a computerised alerting system, which would permit accurate, automated risk assessments in ‘real-time’, which would further support implementation.

## Conclusion

To the best of our knowledge, the MOAT will be the first evidence-based clinical prioritisation tool to identify patients most in need of pharmacists’ input (in terms of their risk of moderate or severe, preventable MRPs) while in hospital. The current research aims to develop a clinically credible and accurate prognostic model. Although further validation will be needed, we believe that the MOAT will have the potential to support pharmacists’ decision making by providing objective assessments of patients’ risk, thereby ultimately improving efficiency and safety.

## Ethics and dissemination

This study has been approved by the Proportionate Review Service Sub-Committee of the NHS Research Ethics Committee Wales REC 7 (16/WA/0016) and the Health Research Authority (project ID 197298). We plan to disseminate the results via presentations at professional, academic and scientific meetings and conferences, and will submit the findings for publication in a peer-reviewed journal, adhering to the Strengthening the Reporting of Observational Studies in Epidemiology guidelines for the reporting of observational studies.^73^ We will also present our findings at relevant patient/public meetings at the study sites, and work with the patient and public members of the project steering group to develop a wider public dissemination strategy.

## Supplementary Material

Reviewer comments

Author's manuscript

## References

[1] Francis R Report of the Mid Staffordshire NHS Foundation Trust Public Enquiry: Mid Staffordshire NHS Foundation Trust Public Inquiry. 2013.

[2] Berwick D A promise to learn – a commitment to act Improving the safety of patients in England. 2013.

[3] The Royal Pharmaceutical Society. Keeping patients safe when they transfer between care providers – getting the medicines right. 2012.

[4] The Royal Pharmaceutical Society. Helping patients to make the most of medicines, good practice guidance for healthcare professionals in England: Medicines Optimisation. 2013.

[5] National Institute for Health and Care Excellence. Medicines optimisation: the safe and effective use of medicines to enable the best possible outcomes. NICE guidelines [NG5], 2015. 26180890

[6] National Institute for Health and Care Excellence. CG138 Patient experience in adult NHS services. 2012. 34260162

[7] van den Bemt PM, Egberts TC, de Jong-van den Berg LT, et al Drug-related problems in hospitalised patients. Drug Saf 2000;22:321–33. 10.2165/00002018-200022040-00005 10789826

[8] Garfield S, Barber N, Walley P, et al Quality of medication use in primary care--mapping the problem, working to a solution: a systematic review of the literature. BMC Med 2009;7:50 10.1186/1741-7015-7-50 19772551 PMC2758894

[9] Lund BC Adverse drug events in older adults: will risk factor algorithms translate into effective clinical interventions? Expert Rev Clin Pharmacol 2011;4:655–7. 10.1586/ecp.11.48 22111849

[10] Krähenbühl-Melcher A, Schlienger R, Lampert M, et al Drug-related problems in hospitals. Drug Saf 2007;30:379–407. 10.2165/00002018-200730050-00003 17472418

[11] Committee of experts on management of safety and Quality in Health Care (SP-SQS) Expert Group on Safe Medication Practices Glossary of terms related to patient and medication safety 2005. http://www.bvs.org.ar/pdf/seguridadpaciente.pdf (accessed Mar 2017).

[12] Pharmaceutical Care Network Europe. The PCNE Classification V 7.0 2016. http://www.pcne.org/upload/files/152_PCNE_classification_V7-0.pdf (accessed Mar 2017).

[13] The Royal Pharmaceutical Society. professional Standards for Hospital Pharmacy Services. Optimising patient outcomes from medicines. 2014.

[14] Stephens M Hospital Pharmacy. 2nd ed: Pharmaceutical Press, 2011.

[15] The Society of Hospital Pharmacists of Australia. Standards of Practice for Clinical Pharmacy Services. 2016.

[16] American College of clinical pharmacy. Definition of Clinical Pharmacy. https://www.accp.com/stunet/compass/definition.aspx (accessed Mar 2017).

[17] Kaboli PJ, Hoth AB, McClimon BJ, et al Clinical pharmacists and inpatient medical care: a systematic review. Arch Intern Med 2006;166:955–64. 10.1001/archinte.166.9.955 16682568

[18] Chisholm-Burns MA, Kim Lee J, Spivey CA, et al US pharmacists' effect as team members on patient care: systematic review and meta-analyses. Med Care 2010;48:923–33. 10.1097/MLR.0b013e3181e57962 20720510

[19] The Royal Pharmaceutical Society. Now or Never: Shaping pharmacy for the future. 2013.

[20] NHS. Five Year Forward View. 2014.

[21] Lord Carter of Coles. Operational productivity and performance in English NHS acute hospitals: Unwarranted variations. 2016.

[22] Nuffield Trust. A decade of austerity? The funding pressures facing the NHS from 2010/11 to 2021/22. 2012.

[23] The King's Fund. Understanding NHS financial pressures - How are they affecting patient care?. 2017.

[24] East & South East England Specialist Pharmacy Services. Prioritising pharmaceutical care delivery at ward level – Vs. 1, 2011.

[25] Health Quality and Safety Commission New Zealand. All hands on deck: prioritisation criteria. 2011 https://www.hqsc.govt.nz/assets/Medication-Safety/Med-Rec-PR/MR-Workshop-2011/MR-Workshop-All-hands-on-deck-Prioritisation-criteria-Nirasha-Parsotam.pdf (accessed Mar 2017).

[26] American Society of Health-System Pharmacists. The consensus of the pharmacy practice model summit. Am J Health Syst Pharm 2011;68:1148–52. 10.2146/ajhp110060 21642580

[27] Dodds LJ Optimising pharmacy input to medicines reconciliation at admission to hospital: lessons from a collaborative service evaluation of pharmacy-led medicines reconciliation services in 30 acute hospitals in England. Eur J Hosp Pharm 2014;21:95–101. 10.1136/ejhpharm-2013-000385

[28] NHS England. Transformation of seven day clinical pharmacy services in acute hospitals. 2016.

[29] Standardise MA Upskill and scale up: how one acute trust is facing the Carter challenge. Pharm J 2016;297:205–7.

[30] Kaufmann CP, Stämpfli D, Hersberger KE, et al Determination of risk factors for drug-related problems: a multidisciplinary triangulation process. BMJ Open 2015;5:e006376 10.1136/bmjopen-2014-006376 PMC4368979 25795686

[31] Joint British Societies recommendations on the prevention of Cardiovascular Disease. Risk Calculator http://www.jbs3risk.com/pages/risk_calculator.htm (accessed Mar 2017).

[32] Waterlow J Waterlow Pressure Ulcer Prevention/Treatment Policy. 2005 http://www.judy-waterlow.co.uk/downloads/Waterlow_Score_Card-front.pdf (accessed Mar 2017).

[33] Urbina O, Ferrández O, Grau S, et al Design of a score to identify hospitalized patients at risk of drug-related problems. Pharmacoepidemiol Drug Saf 2014;23:923–32. 10.1002/pds.3634 24817497

[34] Onder G, Petrovic M, Tangiisuran B, et al Development and validation of a score to assess risk of adverse drug reactions among in-hospital patients 65 years or older: the GerontoNet ADR risk score. Arch Intern Med 2010;170:1142–8. 10.1001/archinternmed.2010.153 20625022

[35] Tangiisuran B, Scutt G, Stevenson J, et al Development and validation of a risk model for predicting adverse drug reactions in older people during hospital stay: Brighton Adverse Drug Reactions Risk (BADRI) model. PLoS One 2014;9:e111254 10.1371/journal.pone.0111254 25356898 PMC4214735

[36] McElnay JC, McCallion CR, Al-Deagi F, et al Development of a risk model for adverse drug events in the elderly. Clin Drug Investig 1997;13:47–55. 10.2165/00044011-199713010-00006

[37] Saedder EA, Lisby M, Nielsen LP, et al Detection of patients at high risk of medication errors: development and validation of an algorithm. Basic Clin Pharmacol Toxicol 2016;118:143–9. 10.1111/bcpt.12473 26299815

[38] Cottrell R, Caldwell M, Jardine G Developing and implementing a pharmacy risk screening tool. Hosp Pharm 2013;71:58–60.

[39] Falconer N, Nand S, Liow D, et al Development of an electronic patient prioritization tool for clinical pharmacist interventions. Am J Health Syst Pharm 2014;71:311–20. 10.2146/ajhp130247 24481156

[40] Roten I, Marty S, Beney J Electronic screening of medical records to detect inpatients at risk of drug-related problems. Pharm World Sci 2010;32:103–7. 10.1007/s11096-009-9352-6 20012362

[41] Hemingway H, Croft P, Perel P, et al Prognosis research strategy (PROGRESS) 1: a framework for researching clinical outcomes. BMJ 2013;346:e5595 10.1136/bmj.e5595 23386360 PMC3565687

[42] Hemingway H, Croft P, Perel P, et al Prognosis research strategy (PROGRESS) 1: a framework for researching clinical outcomes. BMJ 2013;346:e5595 10.1136/bmj.e5595 23386360 PMC3565687

[43] Peat G, Riley RD, Croft P, et al Improving the transparency of prognosis research: the role of reporting, data sharing, registration, and protocols. PLoS Med 2014;11:e1001671 10.1371/journal.pmed.1001671 25003600 PMC4086727

[44] Steyerberg EW, Moons KG, van der Windt DA, et al Prognosis Research Strategy (PROGRESS) 3: prognostic model research. PLoS Med 2013;10:e1001381 10.1371/journal.pmed.1001381 23393430 PMC3564751

[45] Bouwmeester W, Zuithoff NPA, Mallett S, et al Reporting and methods in clinical prediction research: a systematic review. PLoS Med 2012;9:e1001221 10.1371/journal.pmed.1001221 PMC3358324 22629234

[46] Collins GS, Reitsma JB, Altman DG, et al Transparent Reporting of a multivariable prediction model for Individual Prognosis or Diagnosis (TRIPOD): the TRIPOD statement. Ann Intern Med 2015;162:55–63. 10.7326/M14-0697 25560714

[47] Riley RD, Hayden JA, Steyerberg EW, et al Prognosis Research Strategy (PROGRESS) 2: prognostic factor research. PLoS Med 2013;10:e1001380 10.1371/journal.pmed.1001380 23393429 PMC3564757

[48] Hingorani AD, Windt DA, Riley RD, et al Prognosis research strategy (PROGRESS) 4: stratified medicine research. BMJ 2013;346:e5793 10.1136/bmj.e5793 23386361 PMC3565686

[49] Moons KG, de Groot JA, Bouwmeester W, et al Critical appraisal and data extraction for systematic reviews of prediction modelling studies: the CHARMS checklist. PLoS Med 2014;11:e1001744 10.1371/journal.pmed.1001744 25314315 PMC4196729

[50] ClinicalTrials.gov. Development of the Medicines Optimisation Assessment Tool (MOAT) NCT02582463. https://clinicaltrials.gov/ct2/show/NCT02582463 (accessed Apr 2017).

[51] Blix HS, Viktil KK, Reikvam A, et al The majority of hospitalised patients have drug-related problems: results from a prospective study in general hospitals. Eur J Clin Pharmacol 2004;60:651–8. 10.1007/s00228-004-0830-4 15568140

[52] Basger BJ, Moles RJ, Chen TF Development of an aggregated system for classifying causes of drug-related problems. Ann Pharmacother 2015;49:405–18. 10.1177/1060028014568008 25614526

[53] Franklin BD, Birch S, Savage I, et al Methodological variability in detecting prescribing errors and consequences for the evaluation of interventions. Pharmacoepidemiol Drug Saf 2009;18:992–9. 10.1002/pds.1811 19634116

[54] Moons KG, Royston P, Vergouwe Y, et al Prognosis and prognostic research: what, why, and how? BMJ 2009;338:b375 10.1136/bmj.b375 19237405

[55] Moons KG, Altman DG, Reitsma JB, et al Transparent Reporting of a multivariable prediction model for individual Prognosis or diagnosis (TRIPOD): Explanation and ElaborationThe TRIPOD Statement: explanation and elaboration. Ann Intern Med 2015;162:W1–W73. 25560730 10.7326/M14-0698

[56] Dean BS, Barber ND A validated, reliable method of scoring the severity of medication errors. Am J Health Syst Pharm 1999;56:57–62. 10048880 10.1093/ajhp/56.1.57

[57] Rashed AN, Neubert A, Tomlin S, et al Epidemiology and potential associated risk factors of drug-related problems in hospitalised children in the United Kingdom and Saudi Arabia. Eur J Clin Pharmacol 2012;68:1657–66. 10.1007/s00228-012-1302-x 22644343

[58] Schumock GT, Thornton JP Focusing on the preventability of adverse drug reactions. Hosp Pharm 1992;27:538–38. 10118597

[59] Benkirane R, Soulaymani-Bencheikh R, Khattabi A, et al Assessment of a new instrument for detecting preventable adverse drug reactions. Drug Saf 2015;38:383–93. 10.1007/s40264-014-0257-5 25537235

[60] Franklin BD, Reynolds M, Sadler S, et al The effect of the electronic transmission of prescriptions on dispensing errors and prescription enhancements made in English community pharmacies: a naturalistic stepped wedge study. BMJ Qual Saf 2014;23:629–38. 10.1136/bmjqs-2013-002776 PMC4112418 24742778

[61] Royston P, Moons KG, Altman DG, et al Prognosis and prognostic research: developing a prognostic model. BMJ 2009;338:b604 10.1136/bmj.b604 19336487

[62] Katz MH Multivariable analysis: a primer for readers of medical research. Ann Intern Med 2003;138:644–50. 10.7326/0003-4819-138-8-200304150-00012 12693887

[63] Hayden JA, van der Windt DA, Cartwright JL, et al Assessing bias in studies of prognostic factors. Ann Intern Med 2013;158:280–6. 10.7326/0003-4819-158-4-201302190-00009 23420236

[64] Vittinghoff E, McCulloch CE Relaxing the rule of ten events per variable in logistic and Cox regression. Am J Epidemiol 2007;165:710–8. 10.1093/aje/kwk052 17182981

[65] Geeson C, Franklin BD, Wei L Identification of risk (prognostic) factors for medication related problems (MRPs) occurring during hospital admission: a survey of healthcare professionals and patient/public representatives. Int J Pharm Pract 2017;25:40–65.

[66] Peduzzi P, Concato J, Kemper E, et al A simulation study of the number of events per variable in logistic regression analysis. J Clin Epidemiol 1996;49:1373–9. 10.1016/S0895-4356(96)00236-3 8970487

[67] Hohl CM, Yu E, Hunte GS, et al Clinical decision rules to improve the detection of adverse drug events in emergency department patients. Acad Emerg Med 2012;19:640–9. 10.1111/j.1553-2712.2012.01379.x 22687179

[68] Sterne JA, White IR, Carlin JB, et al Multiple imputation for missing data in epidemiological and clinical research: potential and pitfalls. BMJ 2009;338:b2393 10.1136/bmj.b2393 19564179 PMC2714692

[69] Steyerberg E Clinical prediction models: a practical approach to development, validation and updating. Springer 2009.

[70] Sullivan LM, Massaro JM, D'Agostino RB Presentation of multivariate data for clinical use: The Framingham Study risk score functions. Stat Med 2004;23:1631–60. 10.1002/sim.1742 15122742

[71] Altman DG, Vergouwe Y, Royston P, et al Prognosis and prognostic research: validating a prognostic model. BMJ 2009;338:b605 10.1136/bmj.b605 19477892

[72] Moons KG, Altman DG, Vergouwe Y, et al Prognosis and prognostic research: application and impact of prognostic models in clinical practice. BMJ 2009;338:b606 10.1136/bmj.b606 19502216

[73] Vandenbroucke JP, von Elm E, Altman DG, et al Strengthening the Reporting of Observational Studies in Epidemiology (STROBE): explanation and elaboration. PLoS Med 2007;4:e297 10.1371/journal.pmed.0040297 17941715 PMC2020496

