# Supplementary material for: Medicines Optimisation Assessment Tool (MOAT): a prognostic model to target hospital pharmacists' input to improve patient outcomes. Protocol for an observational study
Source: BMJ Open. 2017 Jun 14;7(6):e017509. doi: 10.1136/bmjopen-2017-017509 (PMC5726068; doi:10.1136/bmjopen-2017-017509)
Supplement: Reviewer comments [file bmjopen-2017-017509.reviewer_comments.pdf]

PEER REVIEW HISTORY

BMJ Open publishes all reviews undertaken for accepted manuscripts. Reviewers are asked to complete a checklist review form (**http://bmjopen.bmj.com/site/about/resources/checklist.pdf**) and are provided with free text boxes to elaborate on their assessment. These free text comments are reproduced below.

ARTICLE DETAILS

**TITLE (PROVISIONAL)**	Medicines Optimisation Assessment Tool (MOAT): a prognostic model to target hospital pharmacists' input to improve patient outcomes. Protocol for an observational study
**AUTHORS**	Geeson, Cathy; Wei, Li; Franklin, Bryony

This article was not externally reviewed at BMJ Open. Protocols that have been independently assessed prior to submission to BMJ Open are usually fast-tracked to publication on the grounds that further substantial changes will not be possible. This independent assessment will usually be external, independent review for both a substantial grant award from a non-commercial or government funder and ethics approval.
